# A review and re-interpretation of a group-sequential approach to sample size re-estimation in two-stage trials

**DOI:** 10.1002/pst.1613

**Published:** 2014-04-02

**Authors:** J Bowden, A Mander

**Affiliations:** MRC Biostatistics UnitCambridge, UK

**Keywords:** sample size re-estimation, two-stage trial, conditional power, median unbiased estimation

## Abstract

In this paper, we review the adaptive design methodology of Li *et al.* (*Biostatistics* **3**:277–287) for two-stage trials with mid-trial sample size adjustment. We argue that it is closer in principle to a group sequential design, in spite of its obvious adaptive element. Several extensions are proposed that aim to make it even more attractive and transparent alternative to a standard (fixed sample size) trial for funding bodies to consider. These enable a cap to be put on the maximum sample size and for the trial data to be analysed using standard methods at its conclusion. The regulatory view of trials incorporating unblinded sample size re-estimation is also discussed. © 2014 The Authors. *Pharmaceutical Statistics* published by John Wiley & Sons, Ltd.

## 1. INTRODUCTION

When designing a randomised controlled trial (RCT) to test the efficacy of a treatment in a chosen patient population, assumptions need to be made about the mean and spread of patient responses to treatment in order to derive an appropriate sample size. However, these assumptions may be subject to considerable uncertainty and, if their validity is not subsequently checked, could lead to a hopelessly underpowered or overpowered study. Adaptive designs incorporating sample size re-estimation offer a potential solution to this problem, by enabling interim patient data to be used to decide whether the initial assumptions were sensible and, if necessary, to alter the size and scope of the trial. Methods to update a trial's required sample size using the current estimate of the pooled response's standard deviation are well used and accepted by the regulatory authorities 1, because this does not require unblinding of the treatment and control groups. See for example Gould and Shih 2 and Kieser and Friede 3. Conversely, there has been a poor uptake of methods that allow unblinding to explicitly estimate the difference in response levels across groups, that is, the treatment effect. This is due in part to fundamental concerns over the trial's perceived validity or scientific rigour after unblinding has occurred. However, objections of a more theoretical nature have been raised as well. For example, common methods proposed in this context such as *p*-value combination or variance spending approaches 4,5 can assign unequal weight to patients before and after the sample size re-estimation (SSR). This violates the *sufficiency principle* and is criticised for being inefficient, compared with more established group-sequential methods 6,7. Moreover, the statistical complexity of many methods, and their use of abstract conditional error functions 8 with non-standard critical thresholds, may also serve to discourage their application in real clinical settings.

In this paper, we review a two-stage adaptive design incorporating SSR proposed by Li *et al.* 9. Following the convention of Wang *et al.* 10, we refer to this as the ‘LSW’ approach. We feel that the LSW approach strikes a nice balance between the flexibility of an adaptive design and the rigour of group-sequential design, as well as being comparatively simple to implement. In Section 2, we introduce our notation and describe the motivation for an adaptive SSR design over a fixed sample size design. In Section 3, we introduce the LSW method, show how it can be modified to accommodate capping of the maximum sample size and evaluate the operating characteristics of these two approaches compared with a fixed sample size design. In Section 4, an alternative method for choosing the design parameters of the LSW method is introduced. We conclude with a wide-ranging discussion of the adaptive approach and point to further research in Section 5.

Before proceeding any further, we firstly describe our motivation for this review.

### 1.1. Motivation

Current treatment options for knee osteoarthritis (OA) are not suitable or ineffective for large numbers of patients 11, and surgery is often the only remaining option. A grant application sought funding to conduct an RCT into the effectiveness of a standard rheumatoid arthritis oral therapy (with acceptable toxicity profile) to relieve pain in OA patients. An initial open-label pilot study in patients with knee OA had shown promising results, so the case for an RCT appeared to be strong. However, the funding council decided that the trial's design should be substantially revised. They were concerned in particular about the lack of evidence on the effect size likely to be seen in the RCT context. The funder offered the opportunity to re-submit the application under the proviso that the new trial incorporated an interim analysis, with clear criteria to stop the study or to proceed with the full-scale recruitment (and new sample-size calculation). The authors were contacted through an advisory service to aid in the trial's re-design in light of the funder's response. We were looking for a simple, transparent method that

1. can be fully specified before any recruitment begins;
2. can be understood and easily implemented by an independent data monitoring committee (DMC);
3. is *not* motivated via a complex conditional error function;
4. is implemented through a clear decision framework that links the interim effect size estimate with future sample size via a simple, familiar formula;
5. allows the trial data to be analysed at the end using standard methods;
6. is a practical and understandable alternative to a specific fixed design for funding bodies and trialists to consider.

Despite feeling that the LSW approach satisfied points 1–4 in our checklist, the aim of this paper is to suggest further changes that address points 5 and 6.

## 2. NOTATION AND THE STANDARD SAMPLE SIZE CALCULATION

Assume that observations in the (experimental) treatment group *X* and (standard therapy) control group *Y* are normally distributed with means *μ*_*x*_ and *μ*_*y*_, respectively, and have a common known variance of *σ*^2^. The standardised mean difference, *δ*, is defined as 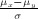 and is the measure of treatment effect we are interested in estimating. An estimate for *δ*, 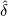, could be obtained from a trial with *n* patients per arm by plugging in an estimate for *μ*_*x*_ and *μ*_*y*_, 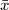 and 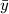, respectively, as shown next:

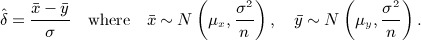


The test statistic, 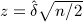, which follows a 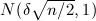 distribution, can be used to test the null hypothesis 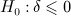. Under *H*_0_, *z* follows a standard normal distribution, so that *H*_0_ is rejected if *z* > *Z*_*α*_, where *Z*_*u*_ = Φ^ − 1^(1 − *u*) and *α* is the type I error rate. In a fixed sample size two-arm trial, the number of participants per arm, *n*, can be determined from the formula:

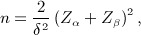


where 1 − *β* is the power reject *H*_0_ at *δ*. For example, to obtain 80% power to detect a difference of 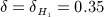 with a one-sided type I error rate of 2.5% (*α* = 0.025 and *β* = 0.2), *n* = 129 patients per arm are needed.

There may, however, be a considerable uncertainty and/or lack of information on the parameter *δ* with which to base this calculation. If *δ* is truly less than 0.35, then substantially more than 129 people would be needed. Equally, if *δ* is truly much larger than 0.35, then the trial may be needlessly large.

### 2.1. A two-stage alternative

Suppose instead that *n*_1_ ( < *n*) subjects are initially recruited per arm into the trial, and an interim analysis is conducted after their responses are observed. This would enable a 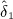 and *z*_1_ to be obtained as

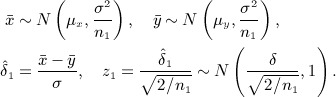
(1)

A decision could then be made on the number of additional subjects per arm needed, *n*_2_ say, in the remainder of the trial, given the magnitude of 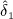 (or equivalently *z*_1_). It is fairly intuitive to see that if one wanted to maintain the same a priori power of 1 − *β* conditional on the value of *z*_1_ (assuming that *z*_1_ is positive), then *n*_2_ would be a decreasing function of *z*_1_—this will be shown explicitly in the next section. However, if *z*_1_ were sufficiently small, then it may be decided to stop the trial altogether; on the grounds that the treatment effect was well short of the clinically relevant difference and, furthermore, the trial would need to be so large to detect this effect that it would be extremely unlikely to attract the necessary funding. Alternatively, the interim data could suggest stopping the trial for positive reasons if 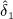 and *z*_1_ were so large as to provide overwhelming evidence against *H*_0_.

However, care must be taken when interim data is used to make decisions about the trial's eventual size and is then subsequently used in the final analysis. Such practices, if unaccounted for, can inflate the type I error rate above the nominal level 8,12. In the next section, we describe the LSW method 9,13 that was proposed for this two-stage design framework. Given an initial sample of patient data, it provides a rationale for deciding whether to stop the trial or continue, and if continuing how many additional patients to recruit. Crucially, it does this whilst controlling the overall type I error rate and also setting the minimum power to reject the null hypothesis conditional on reaching the second stage.

## 3. A REVIEW OF THE LSW METHOD

Suppose that *n*_1_ + *n*_2_(*z*_1_) people are recruited to each arm of the trial according to some as yet unspecified ‘rule’, *n*_2_(*z*_1_), save that *n*_2_(*z*_1_) is 0 when *z*_1_ is less than *h* or greater than *k* (the futility and efficacy boundaries, respectively). Suppose further that if *z*_1_ ∈ (*h*,*k*), then we desire a constant conditional power of 1 − *β*_1_ to reject 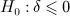 at the trial's conclusion, on the basis of the sufficient statistic and likelihood ratio test:

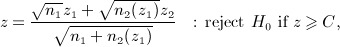
(2)
where *z*_2_ is the test statistic based on *n*_2_(*z*_1_) people, derived in an identical fashion to equation (1). Li *et al.* 9 provide a methodology for choosing the critical value *C* given the design parameters *h*,*k*,*β*_1_ and overall type I error rate *α*, that is independent of the interim test statistic *z*_1_. It can be understood as a simple but clever modification of the general approach of Proschan and Hunsberger 8, which does not share this independence property. The method is now explained in detail using the original notation of Li *et al.* First, define the conditional power function *CP*_*δ*_(*n*_2_,*C* | *z*_1_,*n*_1_) to be

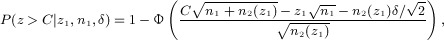
(3)
which is the probability of rejecting the null hypothesis at the trial's conclusion given a sample of *n*_1_ + *n*_2_(*z*_1_) per arm. Let *CP*_0_(*n*_2_,*C* | *z*_1_,*n*_1_) be this conditional power when *δ* is set to 0, so that the null hypothesis is true. The overall type I error rate for the design is equal to

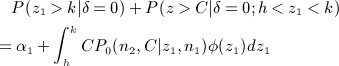

*CP*_0_(*n*_2_,*C* | *z*_1_,*n*_1_) must be between 0 and 1 in (*h*,*k*). Thus, the probability of not accepting *H*_0_ at stage one, *p*^ * ^ say, must be greater than the type I error rate *α*, so that the difference between the two

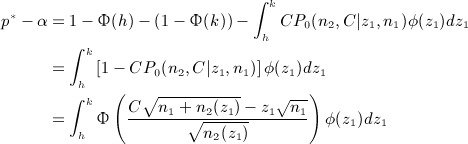
(4)
is the conditional type I error rate in the region (*h*,*k*). From equation (3), if we desire a conditional power of 1 − *β*_1_ to reject *H*_0_ at stage two, then the following must hold:

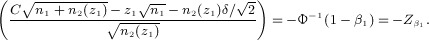
(5)

The two unknowns defining the two stage design are *n*_2_(*z*_1_) and *C*. They could be obtained by solving equation (5) for *n*_2_ (as a function of *C* and *z*_1_), and then plugging in its value to (4) to solve for *C*. Li *et al.* provide a simpler solution; make equation (5) an inequality by replacing 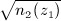 on the denominator with 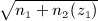, thereby guaranteeing a conditional power of *at least* 1 − *β*_1_. Further substituting *δ* with its stage one estimate, 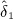 = 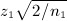, leads to the solution:
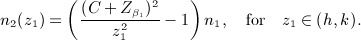
(6)

Note that the equivalent formula to (6) in Li *et al.* 9 is incorrect. Plugging *n*_2_(*z*_1_) into equation (4) and re-arranging yield the following formula for *C*:
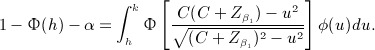
(7)

Equation (7) can thus be solved to yield the critical value *C* needed for the stage two test. Because (7) does not depend on *n*_1_, *n*_2_ or *z*_1_, *C* can be found before the trial starts and any data is observed. Furthermore, as long as *n*_2_ is chosen via equation (6), then any *n*_1_ can be used, meaning the timing of the interim analysis need not be specified in advance. The constant *C* and constant minimal conditional power (set by *β*_1_) of the LSW method are in marked contrast to the original approach proposed by Proschan and Hunsberger. In their approach, a conditional power function 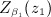 must be specified, and *C* can only be calculated once *z*_1_ has been observed. Note a technical detail; the upper limit on the integral is constrained by the fact that the square-rooted term in the denominator of (7) must be positive, so that 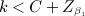. Li *et al.* set the integral's upper limit to 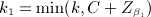 to address this.

The LSW approach's sequential nature, use of pre-specified stopping rules based on sufficient test statistics and its strict control of type I error rate, means that it bears a strong resemblance to a traditional group-sequential trial (GSD). This, we believe, is one of its strengths.

### 3.1. Example: a standard implementation of the LSW method

Following the funder's response to the original arthritis trial application, the LSW method was investigated as a possible alternative. It was decided that it would be feasible to recruit an initial sample of *n*_1_ = 50 patients per arm across the seven study centres within 6 months of the trial commencing. The outcome (change in knee pain from baseline at 24 weeks) would therefore be available for all patients 1 year after study initiation. Trial recruitment would be frozen in this period. If 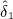 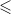 0.2 (equivalent to *h* = 1 on the *z*_1_ scale), then the trial would stop for futility and not recruit any further patients. This would equate to a *p*-value for the null hypothesis 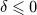 of ≈ 0.16. If on the other hand 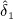 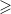 0.55 (equivalent to *k* = 2.76 on the *z*_1_ scale), then the trial would stop for efficacy. This would equate to a *p*-value of 0.003. If however the estimate was between 0.2 and 0.55, then additional participants would be recruited to each arm according to equation (6), guaranteeing at least 80% conditional power to reject *H*_0_, with a type I error rate of 2.5%. By substituting the implied values of *k*, *α*, *β*_1_ and *h* into equation (7), *C* is found (via numerical integration) to be 1.923. The parameters defining this design are listed in Table 1, and it is referred to as ‘design 1’. The value of *k* was chosen to be as large as possible to give the smallest chance of stopping for efficacy at stage one. It is equal to 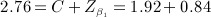. Thus, for the chosen values of (*h*,*α*,*β*_1_), one is forced by the design of Li *et al.* to spend a minimum of 0.003 of the total type I error rate of 0.025 at stage one. However, the futility threshold *h* effectively buys back this type I error rate (and more) because the final threshold *C* is less than *Z*_*α*_ = 1.96.

**Table 1:** Design parameters of the four adaptive trial proposals discussed.

Approach: Design	*h*	*k*	1 − *β*_1_	*C*	*α*	*n*_1_	*n*_*max*_
Standard implementation
LSW: 1	1	2.74	0.8	1.92	0.025	50	333
Modified LSW: 2	1	2.74	0.8	1.93	0.025	50	90
Reverse implementation
LSW: 3	1.14	2.24	0.8	1.96	0.025	70	353
Modified LSW: 4	1.08	2.32	0.8	1.96	0.025	71	121

*n*_*max*_ is the maximum stage 2 sample size.

**Figure 1:**
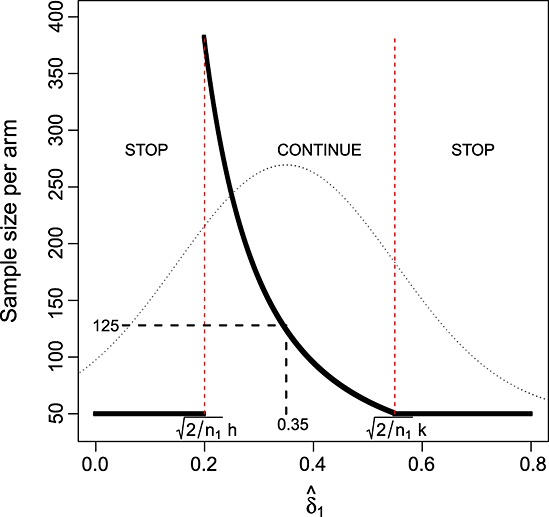
Stage one effect estimate versus total sample size using design 1. Dotted line shows the distribution of the estimate 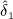 when *δ* = 0.35.

Figure 1 plots the total number of patients needed as a function of 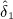 under design 1. We see that at the interim, if 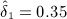, then only 125 patients per arm are required for the trial in total. The dotted line in Figure 1 shows the distribution of the estimate 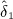 when *δ* = 0.35 to indicate the proportion of times the study would stop early for efficacy or futility at stage one, or continue to stage two with the specified sample size.

### 3.2 Incorporating a maximum sample size constraint

The LSW approach seemed to provide an alternative two-stage trial design that addressed the funders concerns. Although they were not prepared to pay for the fixed trial of 129 patients per arm based on existing evidence, they were potentially prepared to provide full funding if results from an initial phase were sufficiently promising. However, in design 1, the total sample size per arm could be anything from 50 to 383 depending on the value of *z*_1_. Although the research team were keen to define the promising region for *z*_1_ as (1,2.76), it was felt that there would be a maximum sample size (well below 380) beyond which the trial would probably not be funded. A total sample size of 140 per arm, or of *n*_2_ = *n*_*max*_ = 90, was thought a plausible upper limit. To incorporate this constraint into the LSW method and preserve the property that the final stage test statistic threshold is independent of *z*_1_, equations (6) and (7) must be modified. Equation (6) becomes

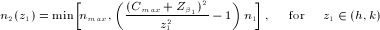
(8)

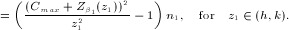
(9)

Although it is convenient to use formula (8) when calculating *n*_2_, we must make use of the equivalent formula (9) in subsequent calculations. It tells us that the constant minimal conditional power term 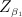 is, in effect, replaced by a simple step function, 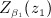, where

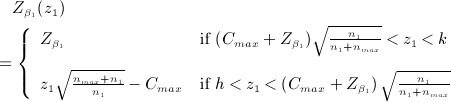
(10)

and the relevant stage two threshold, *C*_*max*_ (which is different to *C*), is found by solving the integral

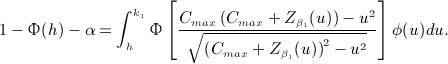
(11)

The upper limit on the integral is defined as before. The denominator of the Φ(.) function in (11) remains well defined for 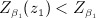 because, from (10), when this occurs

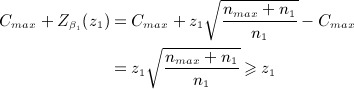


### 3.3 Example revisited

**Figure 2:**
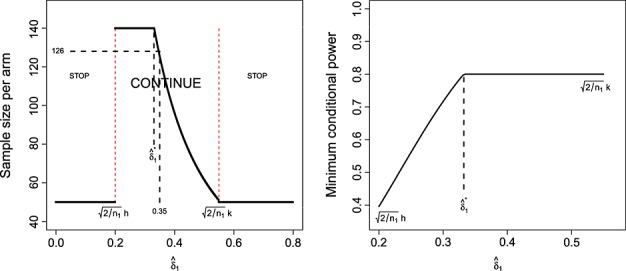
Left: modified LSW design 2. Right: minimum conditional power guaranteed by design 2.

Adding in the extra constraint *n*_*max*_ = 90 to the remaining parameters of design 1 in Section 3.1, we calculate *C*_*max*_ to be 1.936. This is listed as ‘design 2’ in Table 1, and we refer to capping the maximum sample size in this way as the *modified LSW* approach. Figure 2 (left) shows the total sample size of design 2 as a function of 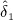. Figure 2 (right) shows the minimum conditional power guaranteed by this design as a function of 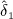. It starts at close to 40% when 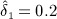 and increases up to a maximum of 80% by 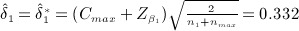. So, artificially constraining the sample size to not exceed a maximum value leads to some loss of power when 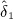 is small. It is therefore important to assess this constraint's effect on both on the overall power and expected sample size of the adaptive trial.

### 3.4. An assessment of designs 1–2

**Figure 3:**
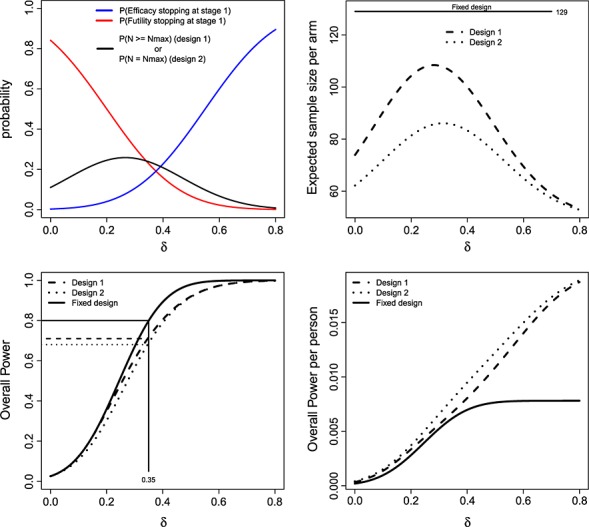
Operating characteristics of the fixed design and adaptive designs 1–2.

Figure 3 highlights the operating characteristics of the original fixed design proposal (*n* = 129, *α* = 0.025, *β* = 0.2) and adaptive designs 1 and 2 as a function of *δ*. They are calculated from the list of expressions given in Table S1 in the appendix (available online as Supporting Information). Figure 3 (top left) shows, for adaptive designs 1 and 2, how the probability of stopping for efficacy or futility changes as *δ* increases from 0 to 1. The two probabilities are equal when *δ* equals the mid-point of 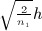 and 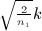. Under design 1, the probability that the total sample size is greater than the maximum of 140 per arm is maximised at around 26% when *δ* equals the mid-point of 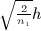 and 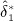. The same value of *δ* maximises the probability that *n*_2_ = *n*_*max*_ under design 2. Figure 3 (top right) shows that the expected sample size of designs 1 and 2 is always less than that of the fixed design. The maximum expected sample size of design 2 is over 20 patients less than that of design 1. Figure 3 (bottom left) shows the overall unconditional power, *P*(Reject *H*_0_), of all three designs. Formula (12) in Table S1 gives this quantity, as well as a more standard formula for the power of the fixed design. The fixed design's overall power is greater than the adaptive designs for all reasonable values of *δ*. At the originally hypothesised value 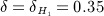, the overall power is 80% by definition, whereas adaptive designs 1 and 2 only achieve an overall power of ≈ 71% and 69%, respectively. This shortcoming of the adaptive designs is returned to in Section 4. Figure 3 (bottom right) shows the ratio of the design's overall power with their expected sample size (which is of course constant for the fixed design). Comparisons of power between designs with different expected sample sizes can be misleading, so the power per unit of expected sample size provides a new and potentially useful standardised measure. Indeed, it has been recently employed by the second author to compare the relative merits of competing development strategies for phase II trials 14. Despite design 2 being the least powerful of the three, it is the superior of the three for all values of *δ* according to this measure. It also highlights how unnecessarily large the fixed design is when *δ* is over 0.5.

## 4. AN ALTERNATIVE IMPLEMENTATION OF THE LSW METHOD

The standard implementation of the LSW approach gives the user freedom to choose the *h* and *k* (albeit subject to some weak constraints) determining whether or not the trial continues to stage two. It also allows the user to specify the minimum conditional power level desired at stage two and leaves the choice of *n*_1_ completely open. As we have seen, this can help identify adaptive designs with a far smaller expected sample size compared with the fixed design. However, because the method is constructed to control the minimum conditional power at stage two, we have also demonstrated that it generally has a lower overall power, as given by equation S1 (available online as Supporting Information), compared with the fixed design. This power loss is especially evident, and indeed relevant, at the originally hypothesised value for *δ*, 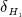. Furthermore, the use of a non-standard critical threshold (*C* or *C*_*max*_) at stage two that is different (and especially lower) than the nominal *α* level will doubtless sit uncomfortably with some trialists. For example, one could envisage the following scenario: a clinical trial using a specific LSW design proceeded to stage two and suggested a rejection of the null hypothesis, because the final test statistic, *z*, was > *C*. However, a standard analysis of the data based around the maximum likelihood estimate (MLE) at the *α* level of significance—which would inevitably be preferred by the trial committee and general medical community—did not. With these two things in mind, we now propose a different rationale for choosing the parameters in an LSW or modified LSW design.

### 4.1 Reverse implementation of the LSW design

For the standard LSW method, rather than choosing 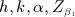 and determining *C*, we instead propose to identify a family of possible designs by implementing the following algorithm:

1. Identify a fixed sample size design with type I error *α* and power 1 − *β* at 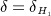.
2. Find all joint values of (*h*,*k*, 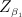) consistent with *α* and *C* = *Z*_*α*_ from equation (7).
3. For each specific value of (*h*,*k*, 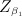), find the minimum value of *n*_1_ that sets the unconditional power in equation S1 at 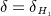 equal to 1 − *β*.

**Figure 4:**
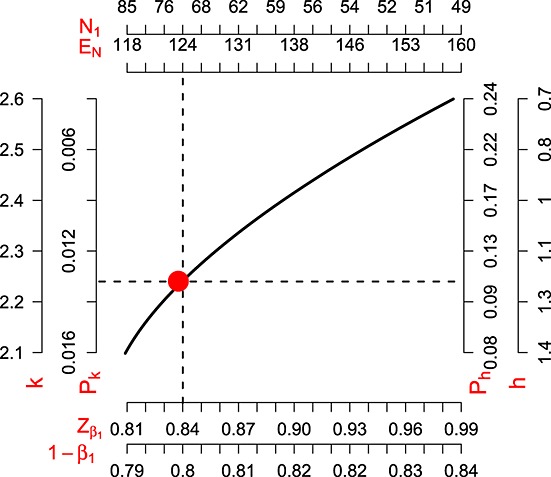
Possible parameter choices under the reverse implementation LSW design.

Fixing *C* to *Z*_*α*_ means that rejection of *H*_0_ at stage two via the adaptive design must coincide with a rejection based on a standard analysis using *z*. The algorithm can be split into the aforementioned steps two and three because equation (7) is independent of *n*_1_, and this also makes the numerical optimisation an easier task. The solid line in Figure 4 shows the values of (*h*, *k*, 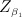, *n*_1_) consistent with this strategy. Scales for *h* and *k* are shown alongside the *p*-values for early stopping due to efficacy and futility (*P*_*k*_ and *P*_*h*_) they imply. For scales 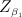 and 1- *β*_1_, the expected sample size per arm at 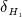 and *n*_1_ are also shown. The red point highlights an interesting and appealing design, where the minimum conditional power equals the unconditional overall power, or *β* = *β*_1_. This occurs at (approximately) *h* = 1.14, *k* = 2.24 and *n*_1_= 70. This is listed as ‘design 3’ in Table 1. The expected sample size at 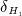 is approximately 123, which is greater than design 1 in Section 3.4 but is still below the fixed design's sample size.

### 4.2 Reverse implementation of the modified LSW design

Our motivation for proposing the modified LSW design was to allow the user to limit the overall trial size through the second stage sample size, *n*_2_, given that the trial had *already* accrued *n*_1_ patients in stage one. However, under the reverse implementation, *n*_1_ is considered as an additional parameter in the design space. This suggests that, rather than simply controlling the stage two sample size via *n*_*max*_, it would be more sensible to control the maximum total sample size per arm, (*n*_1_ + *n*_2_(*z*_1_)). An algorithm to find possible modified LSW designs under this strategy is now described. The algorithm is more computationally demanding than before, because equation (11) depends implicitly on *n*_1_ through 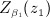.

1. Identify a fixed sample size design with type I error *α* and power 1- *β* at 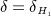. Additionally fix the maximum value of (*n*_1_ + *n*_2_(*z*_1_)), *n*_*Tmax*_ say, and set *C* equal to *Z*_*α*_
2. Given *n*_*max*_ = *n*_*Tmax*_ − *n*_1_, find the joint values of (*h*,*k*, 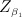, *n*_1_, *n*_*max*_) such that:
  (a) (*h*,*k*, 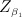, *n*_1_, *n*_*max*_) are consistent with *α* and *C* = *Z*_*α*_ from equation (11).
  (b) *n*_1_ is minimised given the joint values of (*h*,*k*, 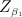, *n*_*max*_).
  (c) The unconditional power in equation S1 at 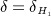 equals 1- *β*.

Table 2 shows the joint values of (*h*,*k*, 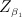, *n*_1_, *n*_*max*_) consistent with this strategy when *n*_*Tmax*_ is fixed at 192 - 50% larger than the original fixed design's sample size per arm of 129. A simple plot is not possible because the design parameters do not all increase or decrease together. We again highlight the design for which the minimum conditional power equals the unconditional overall power, or *β* = *β*_1_. This occurs at *h* = 1.08, *k* = 2.32, *n*_1_= 71 and *n*_*max*_=121 and is listed as ‘design 4’ in Table 1. The expected sample size of the design at 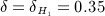 is approximately 111.

**Table 2:** Possible parameter choices under the reverse implementation modified LSW design.

*h*	*k*	1- *β*_1_	*n*_1_	*n*_1_ + *E*[*n*_2_]	*n*_*max*_
0.700	2.76	0.847	54	117	138
0.751	2.66	0.835	56	115	136
0.802	2.59	0.826	58	114	134
0.853	2.52	0.818	61	114	131
0.904	2.47	0.813	63	113	129
0.955	2.41	0.808	65	112	127
1.010	2.37	0.804	67	111	125
1.060	2.34	0.801	70	111	122
**1.080**	**2.32**	**0.800**	**71**	**111**	**121**
1.110	2.30	0.799	72	111	120
1.160	2.27	0.796	75	111	117
1.200	2.25	0.795	77	111	115

**Figure 5:**
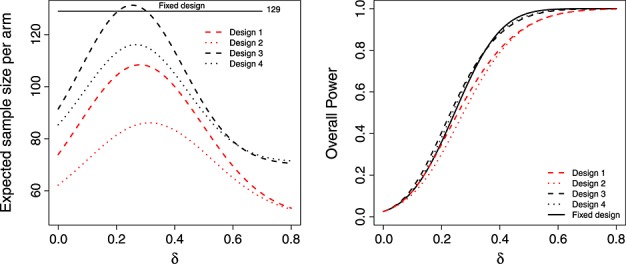
Expected sample size (left) and overall power (right) of the LSW methods under designs 1–4, as a function of *δ*.

Figure 5 compares the operating characteristics four designs featured. Designs 1 and 2 have a far smaller expected sample size than 3 and 4 but because of this, do not control the overall power at 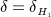 at the original desired level of 80%. As well as being identical to the fixed design at *δ* = 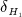, the unconditional power curves of designs 3 and 4 are very close to that of the fixed design across all values of *δ*.

Design 3 has a higher expected sample size than the fixed design at *δ* = 0.35. This may raise concerns in some quarters, but we do not view it as an inherent weakness. Our argument is that funding bodies should generally be prepared to support a trial the size of (or bigger than) the fixed design, but want the chance to stop altogether if the interim data suggests so. This flexibility is provided by the adaptive approach. However, design 4—which additionally caps the maximum sample size—has an expected sample size that is always lower than the fixed design. As is shown in Figure 2 (right), the price paid for this property is that it can exhibit a low conditional power when *z*_1_ is just above the futility threshold *h*.

## 5. DISCUSSION

In this paper, we have reviewed the LSW method for a two-stage trial with SSR and suggested two extensions to make the approach more amenable for use in practice. We firstly modified their standard approach to allow the second stage sample size to be capped. This restriction may be useful in practice, because it would enable funders to maintain a reasonable grip on the maximum cost of the trial when presented with preliminary findings from stage one. The maximum size of the trial may not be limited by the funding body, but instead by the trialists, because of practical constraints on its duration, the number of study centres available and likely recruitment rate. Our modification would also be useful in this case. We secondly suggested an alternative ‘reverse’ implementation of the LSW approach. Our aim was to make the resulting LSW designs a more obvious and transparent alternative to a standard fixed sample size design. The reverse implementation can identify adaptive designs whose unconditional power is near identical with a specific fixed design (across a large range of treatment effects) whilst ensuring that its expected sample size is generally well below that of the fixed design. Furthermore, there is no danger that the resulting decision to reject the null hypothesis at stage two will be odds with inference based around the final test statistic.

### 5.1. The approach of Mehta and Pocock

Although different in its exposition, our approach is similar in spirit to the recent work of Mehta and Pocock 15, that has generated a lot of discussion in the literature (see 16,17). In the same context of two-stage trials with mid-trial sample size adjustment, Mehta and Pocock were motivated to find a simple composite design strategy that would be attractive to trialists who were contemplating moving away from a traditional fixed sample size design. Using the notation of this paper, they encourage the trialist to specify a sample size based on a fixed design (with sample size *n*, type I error *α* and power 1 − *β*), but to allow for an interim after *n*_1_ patients. At this interim, they identify values of the test statistic, *z*_1_, that correspond to so-called *unfavourable*, *promising* and *favourable* regions of conditional power—defined analogously to equation (3). If *z*_1_ is in the *favourable* or *unfavourable* regions then the trialist's are encouraged to continue recruiting up to the original sample size of *n*. However, if *z*_1_ is in the *promising* region, the user is encouraged to increase the sample size (over and above *n* up to a maximum level) and to fix the conditional power at 1 − *β* using the data derived treatment effect estimate. Following this decision framework is guaranteed to not inflate the type I error rate of a standard *α*-level analysis at the trials end. Mehta and Pocock therefore argue that the use of standard analysis methods, as opposed to non-standard adaptive design methods (e.g 4,8), makes the method attractive.

Our reverse implementation of the LSW approach in designs 3 and 4 allow for early stopping at stage one but provide the termination thresholds and a simple SSR rule such that a standard *α*-level analysis is still possible at the final stage. They can deliver substantial reductions in the expected sample size compared with a fixed design when *δ* is much smaller or larger than expected (especially modified LSW design 4), but with no appreciable loss in overall power. Furthermore, the *α*-level analysis conducted at the end of stage two is correct in the sense that it preserves the theoretical type I error rate at *α*, whereas Mehta and Pocock's *α*-level threshold is actually over-conservative and therefore inefficient.

### 5.2. Regulatory support for the use of unblinded SSR

In recent US Food and Drug Administration (FDA) guidance to industry on adaptive trials 1, the FDA is positive about the use of blinded SSR, citing its ability to improve study efficiency and ability to achieve the study goal, without affecting the type I error rate of the trial (Section B, lines 668–674). In contrast, no explicit endorsement can be found in this document on the use of unblinded SSR and so it is necessary to look for principles of guidance more widely.

Revising a study's design in light of an unblinded interim analysis has the potential to induce bias and type I error inflation. Therefore, revisions should be prospectively defined and carefully implemented to avoid bringing the interpretation of study results into doubt. This legitimises the use of GSDs in the FDA's eyes 1 (Section D, lines 817–829), where formal testing of a null hypothesis is carried out at one or more interim analyses to make decisions regarding the continuation of the trial. This is under the proviso that (a) methods for controlling resulting type I error inflation are incorporated as standard; (b) an independent DMC is tasked with reviewing the data at the interim analyses, and furthermore, a statistician independent of the study prepares the report for the DMC.

Throughout this paper, we have pointed the LSW approach's similarity with GSDs, both in their sequential design and in their analysis. So, can current FDA guidance on GSDs be invoked to cover its use? Our opinion is a qualified ‘yes’. Although (a) and (b) would be sufficient for a GSD, the LSW approach (and any SSR procedure for that matter) is arguably more vulnerable to the de-masking of interim results for the following reason: A keen and suitably qualified individual in the study team could potentially transform the recommended stage two sample size into the stage one effect estimate and use this to influence the trial going forward. We therefore believe that an additional condition is necessary; information about the precise value of the stage two sample size must not be fed back to the study team unnecessarily. Rather, trial recruitment should be allowed to continue or stop, until the independent statistician can reveal that the planned size is about to be reached.

### 5.3. Estimation following an adaptive design

The MLE of *δ* at the end of the adaptive design will generally be biased, because it ignores the trial's sequential nature. In the appendix, we provide a detailed investigation of the MLEs properties in this context and contrast it with that of the median unbiased estimate (MUE) suggested by Wang *et al.* 10. The MUE is shown to provide estimates with a reduced bias and mean squared error compared with the MLE, when *δ* is small and positive.

### 5.4. Implementation of the adaptive approach when *σ* unknown

**Figure 6:**
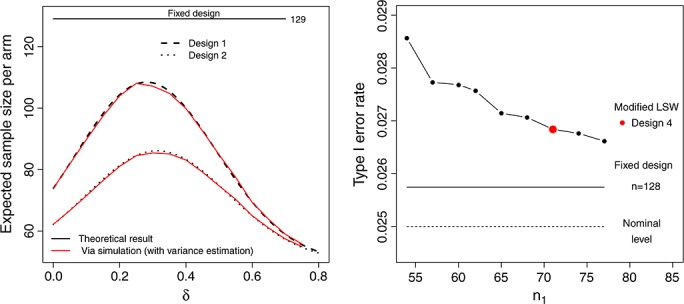
Left: expected sample size of the LSW (design 1) and modified LSW (design 2) using theoretical calculation (black) and using empirical simulation (red). Right: type I error rate inflation (above the nominal 0.025 level) when the data is used to estimate *σ* under the modified LSW design. Design 4 highlighted in red.

We assume that *σ* is known in the calculations used to both find our designs and report their operating characteristics. The simple mathematical formulae would not work if *σ* were treated as a random variable. However, in practice one *will* need to estimate it from the data to implement any of the design proposals. It is important therefore to verify that this estimation does not cause a design's true operating characteristics to differ substantially from its theoretical counterpart. Figure 6 (left) shows the expected sample size of Design's 1 and 2 as a function of *δ* using (a) theoretical calculation (i.e. using formulae from Table S1) and (b) via simulation (incorporating estimation of *σ* separately at stage 1 and 2). To clarify, treatment and control group data were simulated from equation (1) for specific values of *μ*_*x*_, *μ*_*y*_ and *n*_1_, but with a common value of *σ* = 20. This defined the theoretical value of *δ*. A pooled estimate for *σ*, 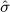 was then obtained from these two populations and 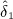 was estimated as 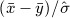. If the trial proceeded to stage 2, *σ* was re-estimated from the *n*_2_(*z*_1_) additionally simulated patients in each arm in the same manner, and used to calculate 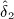, *z*_2_ and *z* for equation (2). The difference between the theoretical expected sample size and those obtained in practice (with estimation of *σ*) is tiny, which is re-assuring. The theoretical and practical power curves for these designs are also near identical (results not shown). However, it is of crucial importance to check that the type I error rate is not drastically inflated (i.e. the power when *δ*=0).

Figure 6 (right) the theoretical and practical type I error rate of the reverse implementation modified LSW design (with a nominal *α*-level of 0.025) as a function of the stage 1 sample size, *n*_1_. Each point corresponds to a row (possible design) in Table 2, with the red dot corresponding to design 4. Some inflation is clearly present. As *n*_1_ increases this inflation reduces and when *n*_1_ is equal to 71 (design 4) the inflation is 0.0018 over the stated 2.5% level. For a comparison we also plot the type I error rate of the fixed design (*n* = 129, *α* = 0.025) when *σ* is estimated from the data. Design 4 is 0.001 above this level.

In summary, there is a minimal difference in the operating characteristics when *σ* is estimated from the data and *n*_1_ is of a reasonable size. It therefore seems a sensible strategy to use the known variance assumption to identify sensible designs and, if one so desires, to then add small perturbations to the parameters in conjunction with empirical simulations until the observed operating characteristics are acceptable. This might be efficiently achieved by substituting threshold parameters (e.g. *h*,*k*,*C*) that are close to the equivalent quantiles mapped from the t-distribution. For example, if the maximum sample size of 192 is used under design 4, then a value for *C*_*max*_ close to *t*_0.025,191_ ≈ 1.972 (instead of 1.96) may be sensible first guess.

### 5.5. Further work

One may wish to extend the modified LSW method to allow not only a maximum cap to be put on the future sample size (given continuation) but also a minimum cap. This extra design facet may be needed in practice if the study team do not wish to halt recruitment whilst waiting for the stage outcome data to be observed. In our motivating example, this was up to 6 months, which is a lengthly delay. Of course, if the interim decision is to stop the trial but further patients end up being recruited, then methods for dealing with trial overrun must be employed. Koyama and Chen 18 have investigated this issue for two-stage trials with a binary response, and it would be interesting to see if this could be generalise to the setting we have discussed.

Using the interim effect estimate to evaluate, the conditional power has been criticised, because for a fixed *δ*, *CP*_*δ*_(*n*_2_,*C* | *z*_1_,*n*_1_) is a random variable containing a substantial amount of variability 19. Thus, it is very important to understand the operating characteristics of any design procedure that utilises conditional power in this way. We have tried to do this here, for a large range of possible values for the parameter *δ*. Unwanted variability in *CP*_*δ*_(*n*_2_,*C* | *z*_1_,*n*_1_) can be mitigated to a certain extent by restricting *n*_1_ to be greater than a minimal value. As we have seen, this will also limit the need to correct for any type I error inflation caused by estimating *σ*. However, as further work, we plan to extend the LSW approach to explicitly account for the uncertainty in the estimation of *δ* and *σ* using Bayesian and semi-Bayesian approaches, as in Wang 20.

Multi-arm multi-stage trials—in which several active treatments are sequentially tested against a standard therapy—are becoming increasingly popular in the era of stratified medicine. The STAMPEDE trial is a prime example 21; it has the additional interesting feature whereby early outcome data (on progression free survival) is used to decide whether specific treatment arms should remain active in the trial, whereas the final analysis of a treatment's effect will be based on overall survival. So far SSR has not been considered for such designs but may offer some utility. To apply the LSW approach, one would need to generalise it to account for interim estimation of the primary endpoint based on a correlated secondary endpoint. The definition of power used to guide the calculation would also need to be carefully chosen, as different definitions are possible when multiple hypotheses are being tested.

Software is made available at http://www.mrc-bsu.cam.ac.uk/Software/download.html to reproduce the set of reverse implementation LSW and modified LSW designs shown in Figure 4 and Table 2 respectively. This work was funded by the Medical Research Council (grant number G0800860).
