# Supplementary material for: A review and re-interpretation of a group-sequential approach to sample size re-estimation in two-stage trials
Source: Pharm Stat. 2014 Apr 2;13(3):163–72. doi: 10.1002/pst.1613 (PMC4288989; doi:10.1002/pst.1613)
Supplement: Supplementary file 1 [file pst0013-0163-sd1.pdf]

Appendix

Operating Characteristics formulae

Quantity	Expression
	Two stage designs $(n_1, h, k, C^*, n_{max}, \alpha, \beta_1)$
$P(z_1 \leq h)$ =:	$\Phi\left(h - \delta/\sqrt{\frac{2}{n_1}}\right)$
$P(z_1 \geq k)$ =:	$\Phi\left(\delta/\sqrt{\frac{2}{n_1}} - k\right)$
$P(n_2 \geq n_{max})$ =:	$\Phi\left((C^* + Z_{\beta_1})\sqrt{\frac{n_1}{n_1+n_{max}}} - \delta/\sqrt{\frac{2}{n_1}}\right)$ - $\Phi\left(h - \delta/\sqrt{\frac{2}{n_1}}\right)$
$E[n_2]$ =:	$\int_h^{k_1} n_2(z_1)\phi\left(z_1 - \delta/\sqrt{\frac{2}{n_1}}\right) dz_1$
$P($Reject $H_0)$ =:	$\Phi\left(\delta/\sqrt{\frac{2}{n_1}} - k\right) + \int_h^{k_1} CP_\delta(n_2, C^*\|n_1, z_1)\phi\left(z_1 - \delta/\sqrt{\frac{2}{n_1}}\right) dz_1$ (12)
	Fixed design (n, α, β)
$P($Reject $H_0)$	$\Phi\left(\delta/\sqrt{\frac{2}{n}} - Z_\alpha\right)$

Table 3: *Expressions for the operating characteristics of the two adaptive trials. C^* should be replaced with C or C_{max} depending on the design. The form of $n_2(z_1)$ also depends on the design*

Estimating δ

Under the two-stage design, the maximum likelihood estimate (MLE) for δ will be

$$\hat{\delta} = \begin{cases} z_1\sqrt{2/n_1} & \text{if } z_1 \notin (h,k) \\ z/\sqrt{n_1 + n_2(z_1)} & \text{if } z_1 \in (h,k) \end{cases} \tag{13}$$

where z is taken from equation (2). Since this completely ignores the sequential nature of the trial it will generally be biased. For this reason, Wang et al.[14] suggest the use of a median unbiased estimator (MUE) in this context. Calculating the MUE requires the design space to be 'ordered' with respect to increasing evidence against H_0. At the point the trial stops, this enables the probability of seeing evidence against H_0 at least as extreme to be specified via a 'p-value function' $P(\delta)$. Wang et al. use the ordering due to Fairbanks and Madsen[16], which is now described. When the trial stops at stage one with a $z_1 \notin (h,k)$ then the only way that the trial could have produced evidence at least as strong against H_0 would be if an outcome $\geq z_1$ had been observed. However,

when $z_1 \in (h,k)$, leading to a continuation to stage two and a final observed statistic, z, then there are two possible ways of observing equal or more extreme evidence: The trial could have stopped at stage one if z_1 had been $> k$. Alternatively, the trial could have proceeded to stage two with any possible value of z_1 as long as an outcome $\geq z$ was eventually observed. Thus, the p-value function can be specified as

$$P(\delta) = \begin{cases} \Phi\left(\delta\sqrt{\frac{n_1}{2}} - z_1\right) & \text{if } z_1 \notin (h,k) \\ \Phi\left(\delta\sqrt{\frac{n_1}{2}} - k\right) + \int_h^{k_1} \Phi\left(\frac{\delta n_2(u)/\sqrt{2} + \sqrt{n_1}u - \sqrt{n_1+n_2(u)}z}{\sqrt{n_2(u)}}\right)\phi(u - \delta\sqrt{n_1/2})du & \text{if } z_1 \in (h,k) \end{cases} \tag{14}$$

The MUE is obtained by finding the value of δ, $\tilde{\delta}$, such that $P(\tilde{\delta}) = \frac{1}{2}$. A two sided (1-$\alpha$) confidence interval for δ can be obtained along identical lines, by finding (in each case) the $\tilde{\delta}$ such that $P(\tilde{\delta})$ equals $\alpha/2$ and (1-$\alpha/2$) respectively. Figure 7 (left) shows a plot of the MUE, $\tilde{\delta}$, under modified LSW design 2.

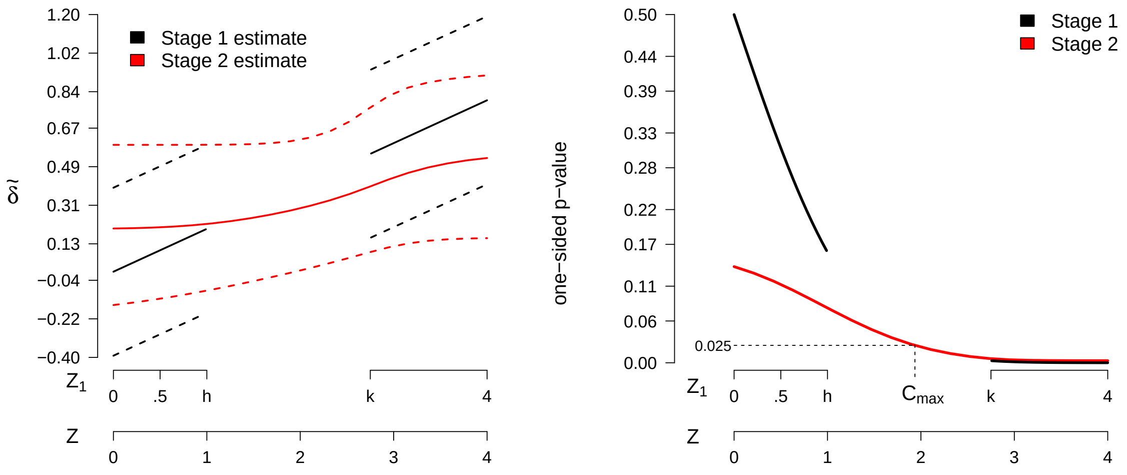

Figure 7: *Left: Median unbiased estimator for δ (plus two-sided 95% confidence interval) as a function of the data accumulated at stage one (z_1) and at stage two z under modified LSW design 2. Right: Corresponding one-sided p-value as a function of the accumulated data.*

When the trial stops at stage one, z_1 is the final statistic and the MUE is trivially equivalent to the MLE, since $\Phi(0) = 1/2$. This is shown by the solid black line, its 95% confidence interval is indicated by dotted black lines. However, the MLE and MUE can differ substantially when the trial continues to stage two. In this case the MLE is a simple function of the combined stage one and two data, encapsulated in the statistic z. For values of z within (h,k) the MLE is given by a straight line joining the two other straight sections (this is not shown). The MUE at stage two is shown by the red line. One can see that, conditional on getting to stage two, the MUE is bounded above (by $h\sqrt{2/n1}$) and below (by $k\sqrt{2/n1}$),

regardless of the value of z. This is a direct consequence of the design space ordering that forces the strength of evidence against H_0 at stage two to be more extreme than if the trial had stopped for futility at stage one ($z_1 < h$) and less extreme than if the trial had stopped for efficacy at stage one ($z_1 > k$). To see this mathematically, note that:

$$P(\delta) \to \Phi\left(\delta\sqrt{\frac{n_1}{2}} - k\right) \text{ as } z \to \infty \quad \text{and} \quad P(\delta) \to \Phi\left(\delta\sqrt{\frac{n_1}{2}} - h\right) \text{ as } z \to -\infty.$$

Figure 7 (right) shows, for the same design, a plot of $P(\delta = 0)$ as a function of the data at stages 1 and 2. This is equivalent to a one-sided p-value for the null hypothesis δ=0. This p-value is consistent with the decision to reject the null hypothesis at stage one if $z_1 \geq k$ and at stage two if $z \geq C_{max}$. Therefore, by design, the p-value function is equal to 0.025 at z = C_{max} and 0.003 at z_1 = k.

Bias and Mean Squared Error

The bias of a generic estimate $\breve{\delta}$ in this setting is given by

$$\int_{-\infty}^{h}(\breve{\delta}(z_1) - \delta)\phi\left(z_1 - \frac{\delta}{\sqrt{2/n_1}}\right)dz_1 + \int_{k}^{\infty}(\breve{\delta}(z_1) - \delta)\phi\left(z_1 - \frac{\delta}{\sqrt{2/n_1}}\right)dz_1 +$$
$$\int_{h}^{k_1}\left\{\int_{-\infty}^{\infty}(\breve{\delta}(z_1, z_2) - \delta)\phi\left(z_2 - \frac{\delta}{\sqrt{2/n_2(z_1)}}\right)dz_2\right\}\phi\left(z_1 - \frac{\delta}{\sqrt{2/n_1}}\right)dz_1$$

For the mean squared error we simply square the error terms within the integrals. They can be evaluated accurately using numerical integration. Figure 8 (left) shows the bias of the MLE ($\hat{\delta}$) and MUE ($\tilde{\delta}$) as a function of δ, for designs 1 and 2. The bias and MSE of the MLE under the fixed design is also shown (the MLE is equivalent to the MUE in a fixed design). The MUE is generally much less biased than the MLE. Figure 8 (right) shows the mean squared error (MSE) for designs 1-4. The MUE also has a smaller MSE than the MLE under across all designs, for δ in the approximate region (0.15,0.45). Although small, this range might be fairly often encountered in practice. By virtue of having larger expected sample sizes than designs 1 and 2, designs 3 and 4 yield estimates for δ with a smaller MSE.

The MLE of the fixed design is unbiased for all values of δ. Furthermore, the MSE of all adaptive designs (regardless of the estimator used) is also substantially larger than the fixed design, which has a constant MSE equal to its variance of 2/129 $\approx$ 0.0155. Straight-forward estimation is therefore much easier within the fixed design.

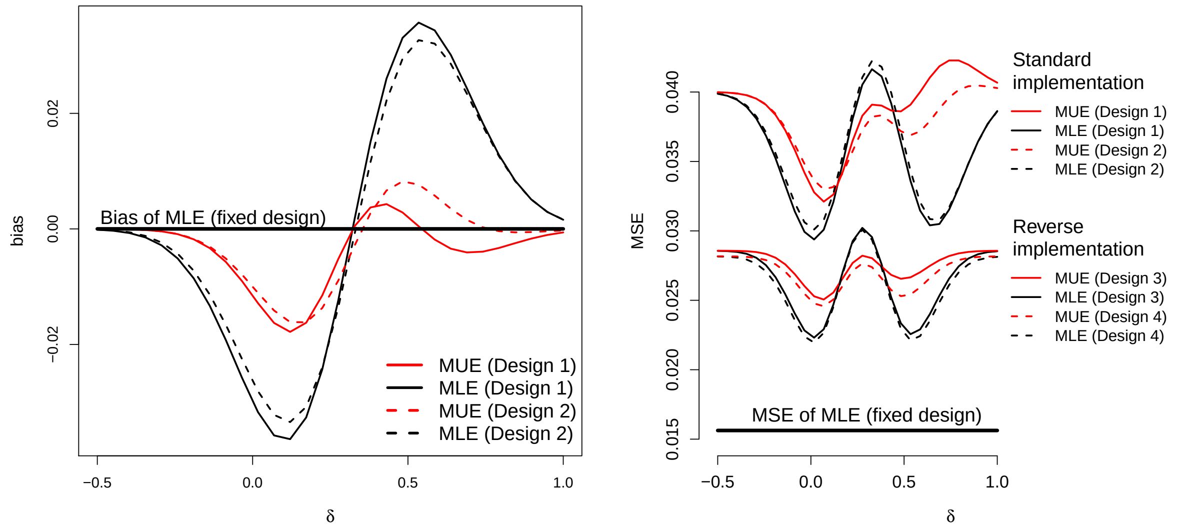

Figure 8: *Left: Bias of the MLE and MUE for designs 1 & 2, as a function of* δ*. Right: Mean squared error (MSE) of design's 1-4, as a function of* δ*.*
